# Corrosion Evaluation of Pure Mg Coated by Fluorination in 0.1 M Fluoride Electrolyte

**DOI:** 10.1155/2021/5574946

**Published:** 2021-05-13

**Authors:** Chun Yu Dai, Xinzhe Gao, ChuanYao Zhai, Qi Jia, Bing Cheng Zhao, HaoYu Shi, Qingting Gao, HongXin Cai, Eui-Seok Lee, Heng Bo Jiang

**Affiliations:** ^1^The Conversationalist Club, School of Stomatology, Shandong First Medical University & Shandong Academy of Medical Sciences, Tai'an, Shandong 271016, China; ^2^Department of Stomatology, The First Affiliated Hospital of Nanchang University, Nanchang, 330006 Jiangxi, China; ^3^Department of Oral and Maxillofacial Surgery, Graduate School of Clinical Dentistry, Korea University, Seoul 02841, Republic of Korea

## Abstract

In the ongoing research on the application of biodegradable materials, surface treatment of is considered to be a relatively effective solution to the excessive degradation rates of Mg alloys. In this study, to further optimize the proven effective surface coatings of fluoride, a low-voltage preparation fluorination method was used to achieve coating effectiveness under safer conditions. Optical observation, scanning electron microscopy (SEM), X-ray diffraction (XRD), energy-dispersive spectroscopy (EDS), and potential dynamic polarization (PDP) experiments were used for the analysis and evaluation. The coating characteristics of the MgF_2_ coatings treated in the 10–90 V voltage range, including the structure, chemical conformation, and electrochemical corrosion assessment, were fully defined. The anodic fluoridation results showed that a pore structure of 1–14 *μ*m thickness was formed on the Mg alloy substrate, and the coating was composed of Mg fluoride. The results of immersion corrosion and electrochemical corrosion experiments showed that compared with pure Mg, anodic fluorinated samples below 40 V exhibited better corrosion resistance, the prepared MgF_2_ coating was more uniform, and the surface mostly exhibited point corrosion. When the voltage reached or exceeded 60 V, the prepared coating exhibited poor corrosion resistance, fracture, and protrusions. After corrosion, it mostly exhibited surface corrosion. The results indicate that idealized coatings can be obtained at relatively low and safe voltage ranges. This finding may enable more economical, environmentally friendly, and safe preparation of coatings.

## 1. Introduction

Mg alloys have been continuously studied in the field of biodegradable materials, because Mg and Mg alloys are the most clinically useful restorative materials owing to their excellent dimensional stability and mechanical properties [1], as well as their reduced potential for stress shielding [2]. Previous studies have discussed both permanent and nonpermanent material designs. In this regard, potential complications and risks of reoperation can be avoided if nonpermanent materials, that is, biodegradable materials, are used [3, 4]. Thus, Mg has been applied in various fields, including biochemistry and materials science [5]. In addition, Mg functions as a cofactor of adenosine triphosphate, induces osteogenesis, and synergizes biocircadian expression, demonstrating its potential as an implant material [6, 7].

However, pure Mg has a limitation for its clinical application and research, which is the fast decomposition rate in humoral situations and the inappropriate corrosion processes that can produce some adverse effects [4, 8–10]. Thus, methods to modulate the dissolution rate of early Mg alloy anodes are required. Therefore, the main objective of our study was to reduce the degradation rate of Mg alloys in a simulated body fluid environment.

Typically, methods to coordinate the alloy corroding behavior include electroplating and surface passivation [11–13]. In addition, ceramic coatings are also effective [14, 15]. By analyzing existing studies, we chose to further optimize the feasibility of the existing fluorination coating treatment [16]. In this treatment, the corrosion conditions and corrosion rates are regulated by insulating the metal from external influences through insoluble corrosion-resistant substances. The metal oxide film formed also has electronic properties such as semiconduction and insulation [17].

Anodization allows the thickness and structure of the prepared coating to be controlled by varying the voltage. However, the prepared Mg oxide coatings can be affected by surface fracture and corrosion resistance [18, 19]. Microarc fluorination (MAF) is a combination of anodic oxidation and fluorination, using high voltage to form a layer of MgF_2_ coating on the sample surface in a fluorinated environment [19–23]. However, the preparation process generates large quantities of hydrogen fluoride gas, which is harmful to humans and causes environmental pollution. Conventional MAF methods generally use voltages above 100 V and high concentrations of hydrofluoric acid [24]. Therefore, this experiment attempted to use a solution with a low fluoride ion concentration as the electrolyte and conduct anodic fluorination (AF) in a lower voltage range. Low-pressure coatings are more environmentally friendly and economical than high-pressure coatings. This reduces the release of irritating fumes, pollution and environmental damage, and harm to humans. The AF process requires a short time. Uniformly dense films can be prepared during this process, and pores are formed. The density of the films is similar to that of bone tissue and has been proven to be highly biocompatible [18, 21–23]. Moreover, the thicknesses of the MgF_2_ coatings prepared under different voltages are different, which is more conducive to cell attachment and subsequent preparation [25, 26]. MgF_2_ has strong corrosion resistance in vitro and can maintain good clinical effects after implantation in the human body [18, 25, 27–29].

In this experiment, the AF technique was used to prepare MgF_2_ ceramic coatings on Mg surfaces. In the fluoride electrolyte (0.1 mol/L NH_4_HF_2_), the coating preparation experiment was performed in the voltage range of 10–90 V. Scanning electron microscopy (SEM) was used to measure the surface morphology of the coating, and potential dynamic polarization (PDP) was used to measure the electrochemical corrosion. The fluorine content of the sample surface under different voltages was analyzed, and the corrosion resistance of the prepared sample in Hank's balanced salt solution (HBSS) was analyzed and evaluated.

## 2. Materials and Methods

### 2.1. Sample Preparation

Pure Mg (Dongguan FeiTai Metal Products Co., Ltd., China) was cut into pieces with dimensions of 20 × 20 × 3 mm. The samples were polished with grade 1200 SiC paper in absolute ethyl alcohol. Then, they were rinsed with absolute ethyl alcohol and blow-dried.

### 2.2. Surface Modification

**Figure 1:**
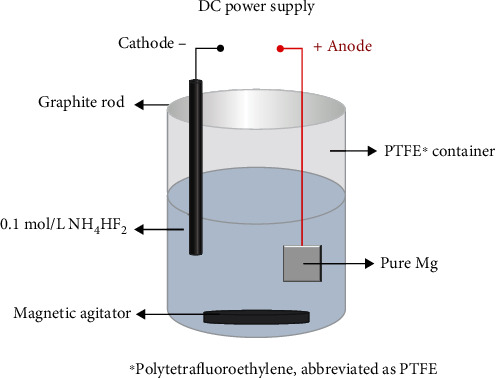
Diagram of AF of pure Mg.

The samples were divided into eight groups and then separately placed in 10, 20, 30, 40, 50, 60, 70, 80, and 90 V electrolytic cells for 3 min as the anode, and a graphite rod was chosen as the cathode. A magnetic bar was chosen to stir the electrolytic solution, which was a solution of 0.1 mol/L NH_4_HF_2_. The progress of the AF is shown in Figure 1. The samples were then washed with distilled water and dried. The conditions of the surface treatment are listed in Table 1.

### 2.3. Surface Characterization

The surface morphologies of the samples and cross-sectional images were observed using a scanning electron microscope (JSM-67000). The elementary compositions were then determined by energy-dispersive spectroscopy (EDS). X-ray diffraction (XRD) measurements of the Mg phase on the surface of the MgF_2_ sample were also performed at a scan rate of 1°/min at 40 kV and 30 mA.

### 2.4. Corrosion Resistance Test

In the immersion corrosion experiment, all processed samples were vertically immersed in the HBSS and kept at 37°C for seven days. For the sample surface area, the volume ratio of HBSS was 20 mL/cm^2^, as per the ASTM standard G31. After seven days, the samples were rinsed with absolute ethanol and blow-dried, and electronic pictures were taken.

### 2.5. Electrochemical Corrosion Test

The exposed fluorinated Mg alloy surface was set to 1 cm^2^. Electrochemical corrosion tests were performed using the PDP method with a constant potentiostat (VersaSTAT 3 : 300) and analyzed using a commercial software (VersaStudio 2.44.4). The classic three-electrode battery consisted of a working electrode for the test sample, a pure platinum rod electrode, and Ag/AgCl/Sat-KCL as the reference electrode (+197 mV compared with a standard hydrogen electrode). HBSS (1000 mL, WELGENE Inc., Korea) was used as an electrolyte and placed in a double-walled beaker, with the temperature maintained at 37 ± 1°C at all times. The scanning rate of the PDP measurements was 3 mV/s.

## 3. Results and Discussion

**Figure 2:**
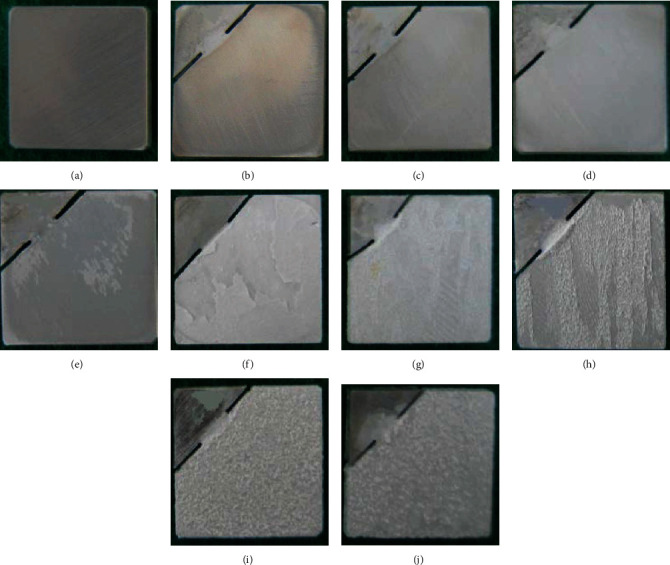
Optical observations of coated samples of (a) pure Mg, (b) AF10, (c) AF20, (d) AF30, (e) AF40, (f) AF50, (g) AF60, (h) AF70, (i) AF80, and (j) AF90.

The surface condition of pure Mg coated by AF can be visually observed in Figure 2. The coating was more uniform before AF50, as shown in Figures 2(b)–2(f). However, there was a large area of coating shedding off the surface at AF50, and different degrees of coating flaring can be found at the shedding junction. The surface topography also became rougher with increasing voltage in the subsequent coating.

**Figure 3:**
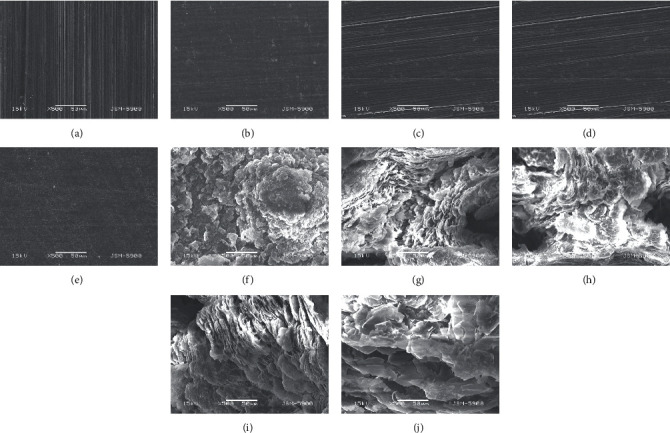
FE-SEM surface morphology of (a) pure Mg, (b) AF10, (c) AF20, (d) AF30, (e) AF40, (f) AF50, (g) AF60, (h) AF70, (i) AF80, and (j) AF90.

Figure 3 shows the varied microscopic surface morphologies of the samples treated at different voltages under SEM. The AF coating started to form at 10 V, and the coating exhibited a dot-like morphology. The AF20 coating appeared more uniformly covered, but the thickness of the coating was not large. The coatings of AF30 and AF40 gradually exhibited a homogeneous matte-like appearance. The coating of AF50 was more differentiated compared to those of AF40 and the previous treatment group. As shown in Figure 3(f), AF50 formed a coating with an inhomogeneous, nondense, and coral-like surface appearance. As the voltage increased, the coral-like shape became coarser and shale-like. The effect of voltage on surface morphology was evident. The surface microstructure was conducive to tissue attachment [30, 31]. The surface morphology of the fluorinated coating prepared in this experiment was slightly similar to that of the microarc oxidation coating, and we speculate that the microgaps and micropores produced on the surface were due to the plasma discharge and the involvement of a small amount of electrolyte particles in the electrolysis [19].

**Figure 4:**
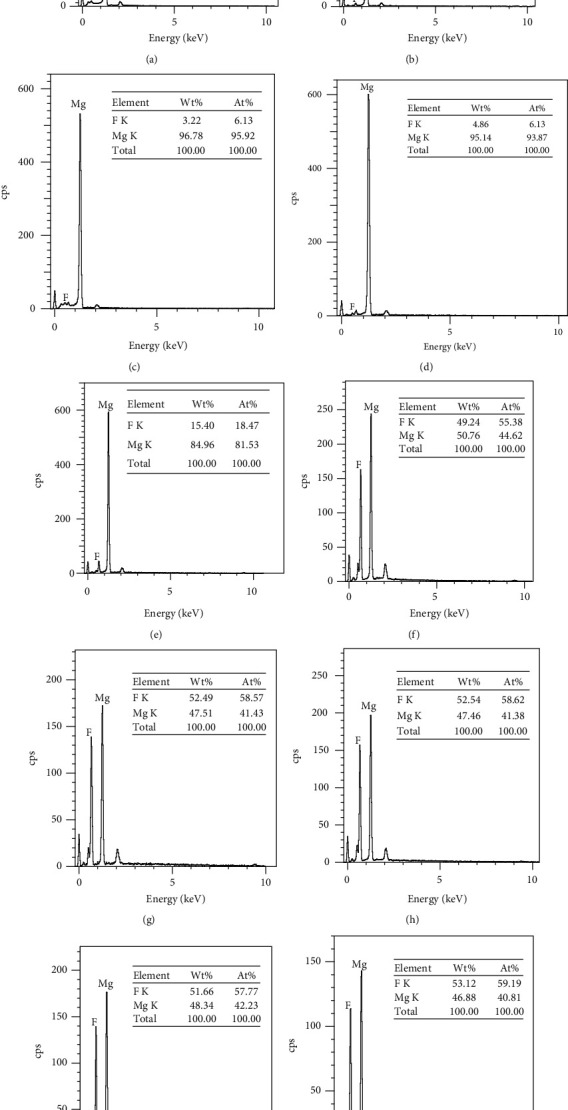
EDS analyses of (a) pure Mg, (b) AF10, (c) AF20, (d) AF30, (e) AF40, (f) AF50, (g) AF60, (h) AF70, (i) AF80, and (j) AF90.

Figure 4 shows the fluoride coating produced on the surface of pure Mg analyzed by EDS. The results show that the F and Mg peaks appeared under different voltages. The main component of the surface coating on the AF sample was MgF_2_. The percentage of elemental fluorine in the coating varied with voltage from pure Mg to 60 V treated samples. The increment in the elemental fluorine content peaked at the 60 V treatment condition, with a 48.28% increase compared to that of the AF10 voltage. The fluorine contents on the surfaces of AF70, AF80, and AF90 were 52.54%, 51.66%, and 53.12%, respectively.

**Figure 5:**
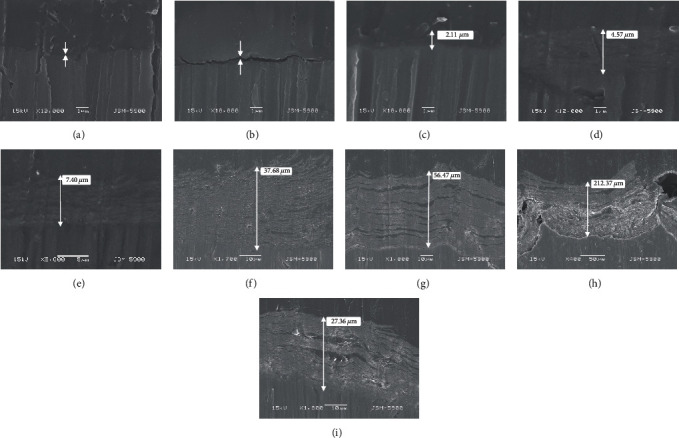
Cross-sectional SEM image of (a) AF10, (b) AF20, (c) AF30, (d) AF40, (e) AF50, (f) AF60, (g) AF70, (h) AF80, and (i) AF90 (the distance between the two arrows above A and B is the thickness of the coating).

Figure 5 shows the SEM cross-sectional morphology of the anodic fluorinated Mg, from which it can be seen that the thickness of the coatings thickened with increasing voltage [17]. The good adhesion of the coating to the Mg substrate without any separation boundary was due to the chemical transformation of the AF coating to the Mg substrate [19].

**Figure 6:**
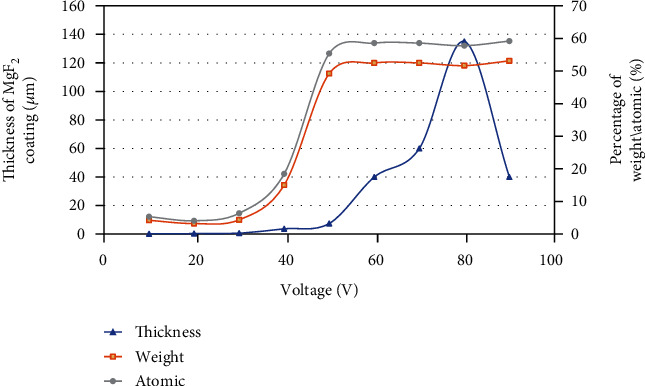
Variation of coating thickness and chemical compositions with processing voltage. In the figure, the blue triangle data point curve corresponds to the variation of coating thickness with the voltage (data from Figure 5). The curves of orange square data points and gray circular data points correspond to the changes of the mass and atomic proportion of fluorine with the increase of voltage, respectively (data from Figure 4).

Figure 6 shows the relationships among the coating thickness, mass, atomic ratio, and voltages obtained from the EDS analysis plotted as a graph. The horizontal axis, left vertical axis, and right vertical axis are the voltage, coating thickness, and fluorine mass to atom ratio, respectively. From the figure, it can be clearly seen that the mass and atomic proportion curves show high consistency as the voltage increases. In the voltage range of 30–50 V, the mass and atomic proportion curves increased at the fastest speed and reached a maximum after 60 V. Because the coating thickness curve rapidly increased after 50 V, EDS X-rays were unable to penetrate the coating and scan for pure Mg, resulting in no significant increase in the mass curve of fluorine and atomic proportion curve. We speculate that the sudden decrease in the AF90 coating thickness may be due to the coating shedding that occurred during the coating preparation process. This also indicates that several microgaps and micropores formed inside the coating at 90 V, which caused the coating structure to be slightly loose and easy to peel off [19].

**Figure 7:**
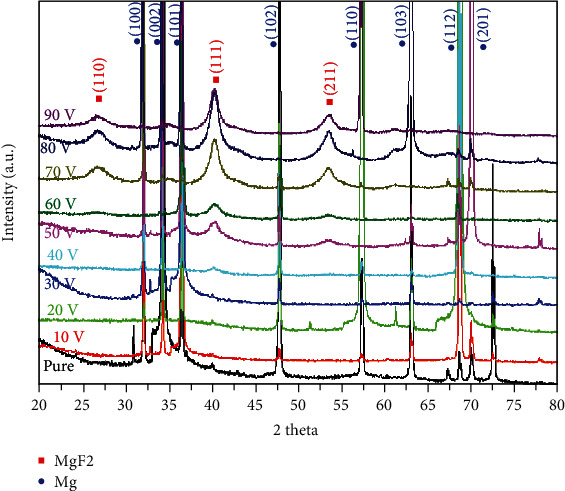
XRD patterns of pure Mg, AF10, AF20, AF30, AF40, AF50, AF60, AF70, AF80, and AF90.

Figure 7 shows the XRD patterns of the pure Mg and MgF_2_ samples. Simultaneously, the crystal structure of the sample surface was analyzed. Compared to the untreated pure Mg, the diffraction pattern of Mg fluoride clearly showed the presence of MgF_2_. As seen in the figure, AF10, AF20, AF30, and AF40 were basically the same in terms of the number of diffraction peaks, angular position, and shape of the diffraction peaks, whereas there were differences in the diffraction patterns of AF50, AF60, AF70, AF80, and AF90. This may be attributed to the change in coating thickness. However, there was only one crystalline phase from AF10 to AF90, which may be because the other crystalline phases were too small or amorphous, as reflected by the XRD analysis [32]. XRD requires a certain thickness of material to be measured to obtain a clear diffraction peak of the sample in the diffraction pattern.

**Figure 8:**
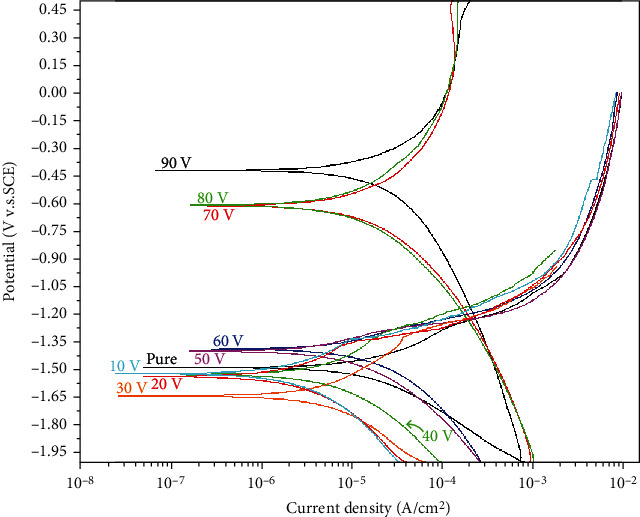
Electrochemical corrosion results: pure Mg, AF10, AF20, AF30, AF40, AF50, AF60, AF70, AF80, and AF90.

To comprehensively and accurately evaluate the corrosion resistance of specimens, it is essential to conduct an electrochemical test on the fluoride-treated object and the control group (pure Mg) [11].  Figure 8 shows the Tafel curves of the samples. The polarization test is an electrochemical method that reflects the corrosion resistance properties by the metal corrosion potential (*E*_corr_) and corrosion current density (*I*_d_). More corrosion-resistant states occur at lower current densities and at relatively high corrosion voltages [2].

As shown in Figure 8, the pure samples exhibited normal corrosion conditions. Therefore, considering the pure Mg characteristics as the evaluation criterion (*I*_d_ pure Mg 2.25 × 10^−5^ A/cm^2^), the electrochemical results were broadly divided into two groups. One group exhibited higher overall current density than the pure Mg (AF50–90, *I*_d_ values of 2.61 × 10^−5^, 3.83 × 10^−5^, 8.55 × 10^−5^, 6.98 × 10^−5^, and 7.72 × 10^−5^ A/cm^2^, respectively), indicating poorer corrosion resistance, which suggests that the coating does not exhibit corrosion resistance effectiveness. On the contrary, the other group exhibited resistance against corrosion, with *I*_d_ values of AF20 4.13 × 10^−6^, AF10 6.37 × 10^−6^, and AF30 7.15 × 10^−6^ A/cm^2^, respectively. Corrosion resistance properties were not obtained at 40 V and higher voltage treatments. However, lower voltages, for example 30 V and below, optimized the corrosion resistance of Mg.

Therefore, a better coating impedance effect occurs in the relatively low-voltage experimental conditions, which is in agreement with the experimental expectations. Moreover, in the low-voltage treatment group, the corrosion resistance was more prominent for AF10, AF30, and AF20 (ranked from highest to lowest). A minimum current density and a relatively high corrosion voltage were obtained for the 10 V treatment sample.

Gu et al. [21] reported that the corrosion resistance of oxide coatings can be modulated by controlling the voltage applied during the coating preparation process. This was confirmed in the present study. In contrast to previous studies [16, 17], in the present experiments, reaction conditions in the safe voltage range were used to prepare coatings with tunable corrosion rates. This further confirms that the application of AF technology to Mg alloy coatings is still promising and valuable for research.

**Figure 9:**
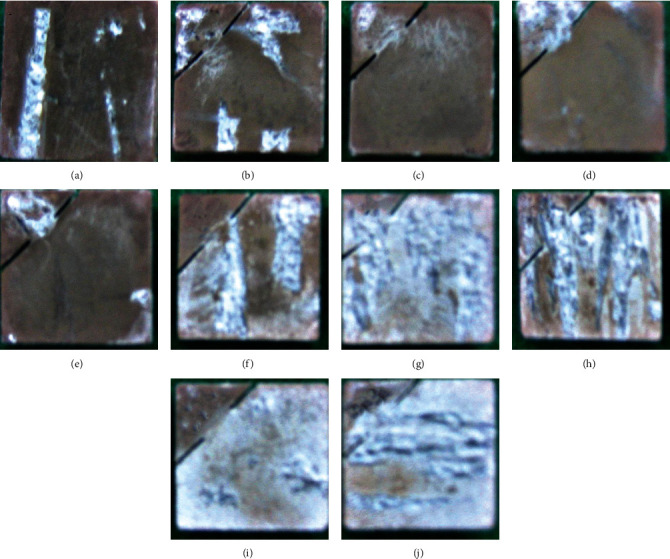
Optical observations of each group of samples after immersion experiments of (a) pure Mg, (b) AF10, (c) AF20, (d) AF30, (e) AF40, (f) AF50, (g) AF60, (h) AF70, (i) AF80, and (j) AF90.

In combination with the SEM results, the fluoride coatings at 50 V and higher showed chipping or spalling, a phenomenon that may lead to uneven distribution under optical observation. According to Hornberger et al., for coating samples, corrosion may start with some defects in the coating, which are nonuniform in nature [33, 34].  Figure 9 shows the optical observation of each group of samples after 7 days of HBSS immersion and etching. During the degradation of the coating, MgF_2_ dissolved into F^−^ and Mg^2+^, and the latter reacted with OH^−^ to produce Mg(OH)_2_ deposits. A layer of Mg(OH)_2_ degradation products covered the sample. Owing to the lower solubility of MgF_2_, Mg(OH)_2_ was generated at a slower rate, and the coating exhibited better corrosion resistance. As corrosion proceeded, H_2_O and Cl^−^ penetrated the Mg alloy matrix, and the Mg alloy started to degrade to form Mg(OH)_2_ and H_2_ [35]. Owing to the absence of acid cleaning, grey-blue surface corrosion product deposits can be observed. Looking at the sediment distribution, we found more uniform observations and a smaller proportion of product distribution in AF20. AF50–AF90 exhibited large product deposits with similar moisture-shedding patterns.

## 4. Conclusion

In this study, under AF treatment, the following conclusions were drawn:

1. After AF treatment, the surface formed coral-like and shale-like surface morphologies
2. XRD and EDS analyses indicated that the main component of the surface coatings formed by AF was MgF_2_
3. Within a certain range, the thickness of the coating increased with an increase in voltage and reached a peak at AF60
4. Compared with the untreated samples, the corrosion resistance of the treated samples was improved. The samples treated at 10 V were more resistant to corrosion

## Figures and Tables

**Table 1:** Sample codes and surface treatment conditions.

Sample code	Treatment conditions	Treatment time (min)
Pure Mg	/	/
AF10	Anodizing by DC^∗^ power supply in 10 V	3
AF20	Anodizing by DC power supply in 20 V	3
AF30	Anodizing by DC power supply in 30 V	3
AF40	Anodizing by DC power supply in 40 V	3
AF50	Anodizing by DC power supply in 50 V	3
AF60	Anodizing by DC power supply in 60 V	3
AF70	Anodizing by DC power supply in 70 V	3
AF80	Anodizing by DC power supply in 80 V	3
AF90	Anodizing by DC power supply in 90 V	3

^∗^DC: direct current.

## Data Availability

The data used to support findings of this study are included within the article.
